# Social phobia and evasiveness: trial protocol for a randomized controlled feasibility and superiority trial of the effect of Modified Collaborative Assessment vs. standard assessment on patients’ readiness for psychotherapy (CO-ASSM-RCT)

**DOI:** 10.1186/s40814-023-01332-z

**Published:** 2023-06-20

**Authors:** Oliver Rumle Hovmand, Nina Reinholt, Kirstine Dichmann, Radoslav Borisov, Sidse Arnfred

**Affiliations:** 1 Department of Clinical Medicine, Faculty of Health, University of Copenhagen, Copenhagen, Denmark; 2 Psychiatric Research Unit, Region Zealand Mental Health Service, Fælledvej 6, 4200 Slagelse, Denmark; 3 Research Unit for Psychotherapy and Psychopathology, Region Zealand Mental Health Service, Fælledvej 6, 4200 Slagelse, Denmark; 4 Department of Forensic Psychiatry, Region Zealand Mental Health Service, Slagelse, Denmark; 5 Psychiatry South, Region Zealand Mental Health Service, Ramsherred 1, 1. Sal, 4700 Naestved, Denmark

**Keywords:** Assessment, Personality disorders, Social phobia, Psychotherapy, Evasiveness, Collaborative assessment, Therapeutic assessment

## Abstract

**Background:**

Evasive personality disorder (AvPD) and social phobia (SP) have substantial costs for patients and their families and great economic costs to the society. While psychotherapy can be an efficacious treatment, many patients drop out during treatment. Increased knowledge on how to decrease dropout from psychotherapy is warranted, including how to increase a patient’s readiness for psychotherapy.

**Methods:**

We describe a randomized controlled feasibility and superiority trial of 42 individuals with a clinical diagnosis of either SP or AvPD, who are to initiate psychotherapeutic treatment in Danish outpatient mental health services. They will be randomized in a 1:1 ratio to either assessment-as-usual and receive no further assessment or to a Modified Collaborative Assessment (MCA) provided as a pre-treatment intervention before psychotherapy initiation. MCA will include a battery of psychological tests designed to thoroughly assess the patients’ psychopathology. The tests are administered in collaboration with the patient, including detailed oral and written feedback. We hypothesize that the intervention is feasible regarding patient’s acceptance and adherence. We further hypothesize that patients randomized to MCA will reach higher levels of readiness for psychotherapy as assessed with the University of Rhode Island Change Assessment Scale (URICA).

**Discussion:**

This protocol assesses the feasibility, efficacy, acceptability, and safety of an intervention aimed at changing the readiness for participation in psychotherapy of patients with SP and AvPD. Results from this feasibility study could guide the development of future large-scale trials of MCA and procedures for MCA treatment fidelity assessment.

**Trial registration:**

NCT2021001.

## Background

### Introduction and rationale

Anxiety disorders represent an important public health concern in the Western world [1, 2]. Estimates from a large European epidemiological survey suggest that 14% of the European population will meet the criteria for an anxiety disorder within their lifetime [3]. These disorders are often associated with a chronic, debilitating course for the affected individual as well as high socio-economic costs [4–6]. Anxiety disorders are among the leading causes of the global disease burden and the annual costs in Europe alone reached 74 billion Euros in 2010 [7, 8]. Continuous efforts to improve treatment programs for anxiety pathology are imperative.

Social phobia (SP) is the most common among the anxiety disorders. The fear of being observed or negatively evaluated by other people is a prominent characteristic of individuals with social phobia. This fear leads the individual to avoid performance or social situations (e.g., speaking or eating in front of others, making acquaintances, and meeting authorities), or enter such situations with substantial discomfort [9]. This evasiveness severely impacts the social functioning and quality of life of affected individuals [10, 11].

Similarly, avoidant personality disorder (AvPD) is characterized by a pervasive pattern of social inhibition, feelings of inadequacy, and hypersensitivity to negative evaluation, which result in marked evasiveness and avoidance of social interactions. AvPD patients perceive themselves as unwanted and isolated from others [9]. The Diagnostic and Statistical Manual of Mental Disorders, fifth edition (DSM-5) [12], recognizes a considerable overlap between AvPD and SP. Although the relationship between the disorders is a matter of debate [13], the dominant conceptualization is that the two disorders represent a spectrum, differing from each other only in severity (the severity continuum hypothesis [14]). In the upcoming revision of the International Classification of Diseases, tenth edition (ICD-10), AvPD will be removed as an independent diagnosis [15], which further supports the severity continuum hypothesis. Similar to the anxiety disorders, AvPD is associated with profound impairment in daily life for the affected individual, as well as high socio-economic costs [16]. Hence, the present study protocol relevantly focuses on SP and social evasiveness.

Danish outpatient mental health services provide time-restricted, standardized, interdisciplinary treatment programs for social phobia and AvPD. Following national clinical practice guidelines, the treatment programs offer evidence-based cognitive-behavioral therapy for social phobia and mentalization-based therapy for AvPD.

The content and format in the standardized treatment programs for moderate-severe SP and AvPD are regulated in accordance with the Danish Health Authority guidelines. The treatment program for SP consists of diagnostic assessment (1–3 consultations), medical consultations (1–2 consultation), and group therapy with two therapists (14 sessions (120 min)), relatives’ support (1 session), and network consultation (1 session) [17]. Correspondingly, the standardized outpatient treatment program for AvPD includes up to 78 h of clinician time consisting of clinical assessment (2 h), medical consultations (1–2 consultations), group therapy with two therapists (30 sessions (120 min)), and network consultation (1–2 sessions) [18].

However, despite a solid evidence base for the efficacy of cognitive-behavioral therapy for social phobia, recent meta-analytic data suggest that only 45% of patients suffering from social phobia remit from their principal diagnosis after treatment and patients with social phobia have a worse outcome than patients with other anxiety disorders [19]. The evidence-based psychological treatment for avoidant personality is limited in terms of number and quality of studies and the remission rates vary substantially from 40 to 80% [20].

Data from a recently finalized multicenter randomized controlled trial [21] investigating the relative efficacy of group diagnosis-specific versus transdiagnostic cognitive-behavioral therapy for anxiety disorders or depression supports the meta-analytic findings on social phobia. In this trial, 291 patients with anxiety disorders or depression received standardized treatment programs from three Danish mental health services, and the results suggested that only half of the patients no longer met diagnostic criteria for their principal diagnosis by the end of treatment [22]. No data exists on the efficacy of the standardized programs for AvPD.

### Modified collaborative assessment

Psychiatric assessment usually aims to establish a diagnosis and plan the treatment, but it is not considered part of the treatment proper. We wish to alter this perspective by the introduction and exploration of a modification of Collaborative Assessment that we have chosen to name Modified Collaborative Assessment (MCA).

MCA takes off from Collaborative Assessment and Therapeutic Assessment (C/TA) [23–25]. These terms are used to describe a family of semi-structured, brief therapeutic interventions, in which a therapist administers a large battery of standardized diagnostic and psychological tests in collaboration with a patient and delivers feedback in a manner that is useful and enriching—and therefore therapeutic—for the patient.

C/TA has been explored in several controlled trials with adults and has been shown to increase a range of process variables related to therapy outcomes. This includes self-esteem [26–29], compliance with treatment recommendations [30], therapeutic alliance with subsequent therapist [31, 32], and satisfaction with treatment [33], as well as decreased anxiety symptoms [34, 35] and levels of self-criticism [34]. In addition, Poston and Hanson [36] published a meta-analysis on 17 published C/TA studies, which found favorable effects of this intervention in terms of overall effectiveness when compared to assessment as usual.

We wish to apply a modification of C/TA, where the intervention is shorter, is slightly more structured, and requires less psychiatric expertise (i.e., it can be carried out by trainee doctors and psychologists), which we therefore expect to be more feasible in the trial as well as in later implementation.

Like C/TA, MCA will include the administration of standardized diagnostic instruments, but in contrast to C/TA, we will only include a smaller selection of tests in order to secure feasibility. The battery of tests will be specifically designed to gather information on psychopathology which a brief clinical interview might not detect, such as symptoms of previously undetected developmental disorders or incipient psychosis. We have compiled a battery of tests with this focus because we find it most suitable for application in the Mental Health Service. The present study is further designed to establish diagnosis adhering to the current diagnostic systems (ICD-10 and DSM-5), but we expect it would also be applicable if the Mental Health Service introduced the dimensional [37, 38] model of psychopathology, since the current MCA also includes a thorough personality assessment according to the DSM-5 alternative model of personality pathology.

MCA emphasizes respect for the patients as “experts on themselves.” The assessor will, in collaboration with the patient, formulate a list of therapeutic questions which the patient would like to “ask the psychological tests.” This will help guide the patient and assessor’s collaborative quest to learn more about the patient’s problems and personal resources. The results of the assessment and the answers to the therapeutic questions will be communicated, respectfully, to the patient both orally and in writing. It will further be communicated to the patient’s future therapist in writing. In this manner, it should be possible to formulate personally relevant problems for later psychotherapy. The MCA assessor recognizes that diagnostic assessment is an interpersonal event and that the relationship between assessor and patient is paramount both in relation to the validity of the result and the patient’s further treatment [25].

In short, MCA is a brief, individualized, and person-centered assessment of psychopathology, where assessment, psychotherapy, and psychoeducation are integrated into a novel intervention, all carried out in collaboration with the patient.

### Readiness for psychotherapy

The fundamental role of patients’ readiness for psychotherapy change (or client motivation) in the outcome of therapy is widely recognized [39]. The overall concept refers to the intentional aspect of change, the internal drive preceding behavioral change before the initiation of therapy, and the ongoing engagement throughout therapy [40]. Theoretically, the concept is most profoundly described as a core component in the “stage of change” dimension of the so-called Transtheoretical Model of behavioral change set forward by Prochaska and DiClemente [41]. In the “stage of change” dimension, patients are assumed to vary in their overall readiness to change, being on different levels ranging from “pre-contemplation” to being ambivalent about change (“contemplation”), having intentions to change (“preparation”), starting changes (“action”), and consolidating changes (“maintenance”).

Studies have consistently found patients’ readiness to change to be an important factor in predicting and moderating their psychotherapy outcomes [42]. Regarding anxiety disorders, research indicates that patients’ readiness to change reduces symptoms and improves other process variables, such as working alliance and adherence to treatment [43]. However, data suggests that up to 80% of patients are not ready for change (to pursue treatment goals) when they enter treatment and possess ambivalence about therapy [44].

We expect that MCA will increase patients’ readiness for psychotherapy, as assessed by the University of Rhode Island’s Change Assessment Scale (URICA) (contemplation subscale) and the Readiness for Psychotherapy Index (RPI), and will increase engagement in psychotherapy as measured by attendance. We expect that more than one mechanism of action is at play: (a) the patient will develop a relationship with the MCA accessor and the outpatient clinic during the course of MCA, which will carry over to the therapeutic relationship with the psychotherapist; (b) due to the structural MCA format, the patient will be confident that her problems are seen and understood; (c) the patient will understand herself, her problems, and personal strengths and will be able to work more effectively on these in therapy; and (d) the therapists will have a greater knowledge of the patient’s problems based on the summaries from the MCA.

### Objectives

The study objectives are to (1) explore the feasibility of MCA as an intervention through rates of recruitment of screened patients, patience’s adherence to MCA or Assessment As Usual (AAU), patient satisfaction ratings, and patient and therapist/clinician evaluations; (2) compare the effect of MCA versus AAU on levels of readiness for psychotherapy in patients referred to group therapy for social phobia or AvPD at end-of-intervention (T1) (main outcome) and after 1-month follow-up (T2); (3) compare the effect of MCA vs AAU on diagnoses (number of diagnostic revisions) and treatment offered (number of patients offered other or additional treatment) in patients referred to group therapy for social phobia or AvPD, as well as adherence to group therapy within the first 4 weeks; and (4) develop a fidelity checklist for the MCA intervention.

We hypothesize that MCA is feasible regarding acceptance and adherence in patients with social phobia or AvPD and is superior to AAU in increasing contemplation score (URICA, see below) at the end of intervention (T1). (2) In addition to this, patients offered MCA have higher service satisfaction ratings (CSQ) than those offered AAU prior to psychotherapy onset, and user evaluation scores of MCA (purpose-made) are positive (more than 3 on a 1–5 Likert Scale).

## Methods

### Design

The present protocol is based on reporting guidelines from the SPIRIT guideline for Standard Protocol Items for Clinical Trials [45] adapted as recommended when reporting protocols of feasibility trials [46]. The study is designed in order to inform and evaluate the feasibility of a possible future, full-scale RCT of MCA versus AAU. We will evaluate the feasibility of the interventions in terms of clinician resources and patient’s acceptability and adherence to the intervention and comparator, selected based on recommendations for the conduct of feasibility trials [47–49].

In addition to these feasibility outcomes, we will assess outcomes regarding readiness for change, symptoms, and regarding self-esteem and self-efficacy. Data are gathered from patients through a number of questionnaires delivered by the RedCap © webmodule prior to randomization (T0), at the end of MCA (T1) and after 4 weeks of psychotherapy (T2)—the absolute time depends on clinical logistics and timing of group therapy onset.

### Trial design

A two-armed, parallel randomized controlled feasibility and superiority trial comparing the effect of pre-treatment MCA with AAU.

**Fig. 1:**
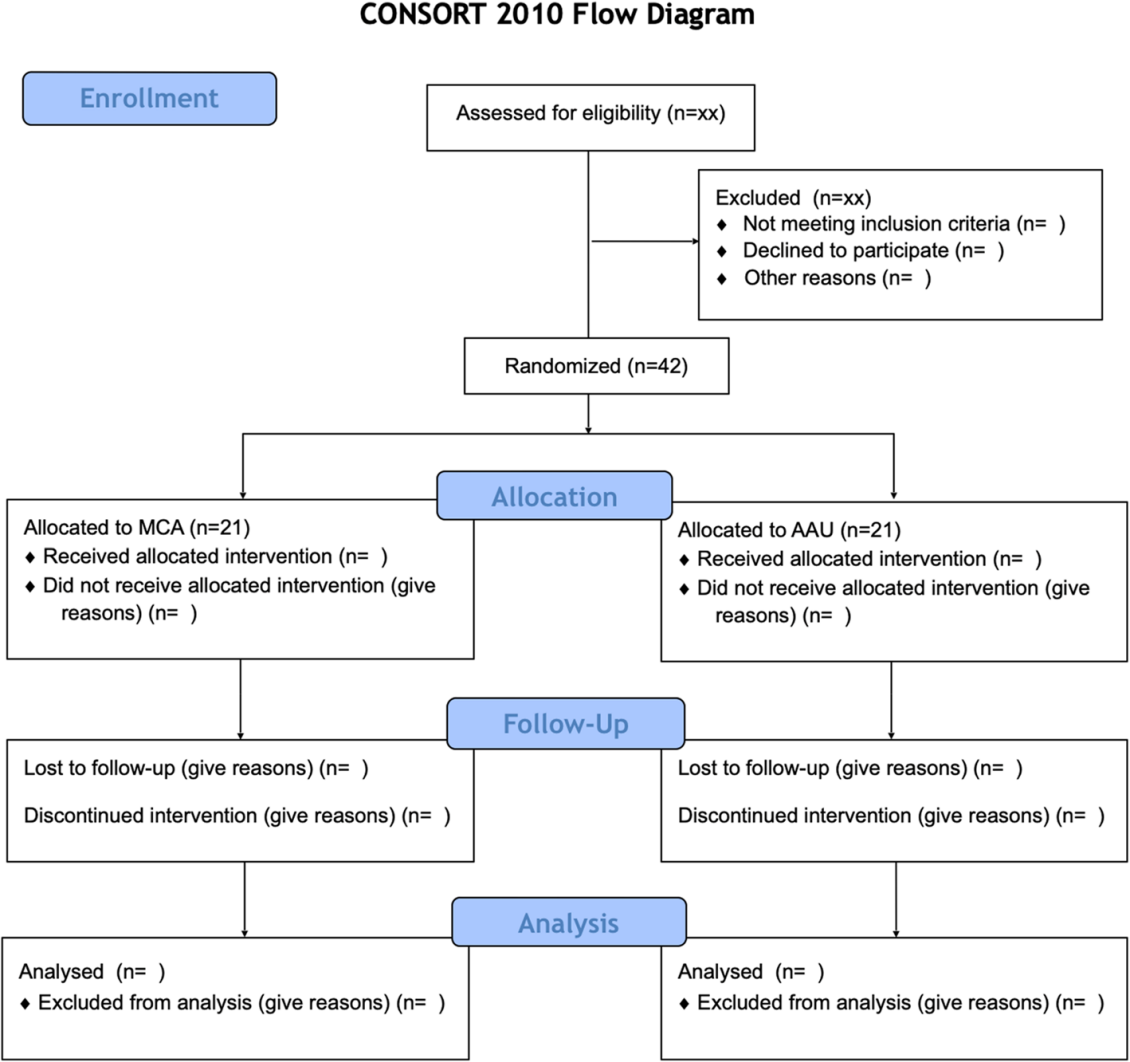
CONSORT 2010 flow diagram

**Fig. 2:**
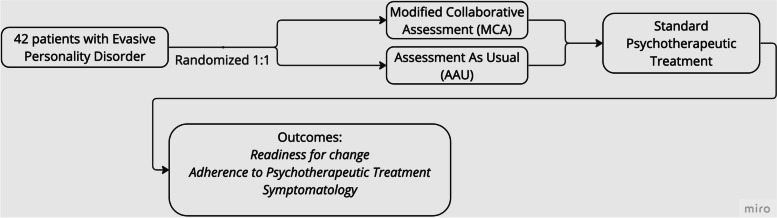
Flow diagram of the intervention

A CONSORT diagram is provided in Fig. 1. A diagram of the proposed study and the outcome assessment is provided in Fig. 2. The trial data collection and randomization, stratified by gender, will be carried out in the web-based data management system REDCap (https://www.project-redcap.org/). Self-ratings will also be collected on the web-based REDCap platform.

### Setting

The setting of the study is Psychiatry South in Region Zealand, which is a rural region with several medium-sized cities, according to Danish standards. Three of these cities have psychiatric outpatient clinics (Maribo, Slagelse, and Naestved), which carry out psychotherapeutic treatment of patients with emotional disorders too serious to be managed by family doctors and primary sector psychologists and psychiatrists. Patients are typically referred by general practitioners, when they have failed to respond to one or two different treatments (medication and/or psychotherapy). The services in these clinics are covered by Danish public health insurance and involve both psychotherapy and psychopharmacological treatment (see also the “Introduction and rationale” section).

### Participants and eligibility criteria

We aim to include 42 patients who satisfy the inclusion criteria: (1) a patient with a tentative ICD-10 diagnosis of either SP or AvPD, (2) who is going to be offered treatment in the aforementioned clinics, (3) is 18–65 years of age, (4) has given written consent to participate, and (5) has sufficient knowledge of the Danish language.

Patients will be excluded if (1) the risk of suicide is high or moderate according to the investigator, (2) they have alcohol or drug dependency, and (3) they have a co-occurring eating disorder with BMI < 18 or psychotic illness.

### Recruitment

In the first consultation at the psychiatric clinic, clinicians will evaluate if the patient is eligible for psychotherapeutic treatment and stipulate a clinical diagnosis. If patients are eligible for treatment in the clinic, they will be invited to an information meeting with the first author, where they will be provided with information about the project. Following this, they can give informed consent to participate.

### Randomization and blinding

Patients will be randomized 1:1 to either the MCA or AAU on an individual level. Allocation to experimental intervention or comparison intervention will be computer-generated using the software REDCap © [50].

Due to the nature of the intervention, neither participants nor the researcher who will administer the intervention can be blinded to allocation. However, data will be re-coded for concealment and analyzed without access to information about allocation. The conclusion will be written prior to unblinding.

### Experimental intervention

The MCA, as described in the “Introduction and rationale” section, will include the possible administration of the following nine assessment instruments:

### Present State Examination (PSE)

PSE is a semi-structured interview that seeks to provide an objective evaluation of symptoms associated with mental disorders. It consists of 140 items and is scored on a 3-point or 4-point scale [51].

### Structured Clinical Interview for DSM-5 (SCID-5)

SCID-5PD is a semi-structured interview guide for evaluation of the 10 DSM-5 Personality Disorders.

### The Examination of Anomalous Self-Experience (EASE)

EASE is a semi-structured checklist for clinical-phenomenological exploration of experiential disturbances. Scores are summed up in a *global score*, with five *sub-scores*: Cognition and stream of consciousness, Self-awareness and presence, Bodily experiences, Demarcation/transitivism, and Existential reorientation [52].

### The Screen for Cognitive Impairment in Psychiatry (SCIP)

SCIP is a neuropsychological test for quick and objective quantification of cognitive function in patients with psychiatric disorders. The Danish translation has demonstrated validity for the detection of objective cognitive impairment [53]. It assesses verbal learning and memory, delayed memory, working memory, word mobilization, and processing speed [54].

### Autism Diagnostic Observation Schedule (ADOS-2)

ADOS-2, module 4 [55], is a semi-structured and standardized observation of communication, social interaction, and creative use of materials used to assess autism spectrum disorder pathology.

### Wechsler Adult Intelligence Scale—Fourth Edition (WAIS-IV)

The WAIS is an IQ test designed to measure intelligence and cognitive ability in adults and older adolescents [56]**.**

### Conners’ Adult ADHD Rating Scales (CAARS)

The CAARS is a test developed to diagnose attention problems, such as ADHD and ADD. It provides both Self-Report and Observer Report Forms, permitting multimodal assessment of adults with attention problems [57].

### Level of Personality Functioning—Brief Form 2.0 (LPFS-BF)

LPFS-BF is a brief 12-item self-report inventory developed to assess levels of personality functioning, as defined in the alternative model for personality disorders in DSM-5 Section III. It measures impairment in personality functioning within the domains of self-functioning and interpersonal functioning [58].

### Personality Inventory for DSM-5, 36-item version (PID-36)

The PID-36 is an abbreviated version of the originally 100-item version of the Personality Inventory for DSM-5 (PID-5), which was developed to measure the pathological trait specifiers listed in the alternative model for personality disorders in DSM-5 Section III [59].

The instruments will be administered by the first author. No other clinicians or therapists will see the included patients in this period of time. Materials from the medical record and from the full MCA will be presented for case supervision with a senior psychiatrist, with the option of getting an additional opinion from another senior consultant in case of diagnostic uncertainty. This procedure is included in order to ensure solid diagnostic verification or alteration. Therapists and teams are informed of the results of the MCA, in order for them to use the extra information about the patient in the following psychotherapeutic intervention, which will be administered after completion of the primary endpoint.

### Comparison intervention

Patients allocated to the control group will receive AAU, which is the standard assessment that patients receive in the clinic, administered as follows: the patients are referred to the clinic by a family doctor or a private practice psychiatrist. On intake to the clinic, they will receive a diagnostic interview with either a medical doctor or a psychologist which can be supplemented with specific psychological tests, (e.g., SCID-5) when personality disorders are suspected, depending on the current resources and competencies in the clinics and following request from the head of treatment. Supplementary assessment is indicated roughly in one case out of ten. Otherwise, AAU patients are on the waitlist for the group therapy and are not followed by any therapist. Following the end of the MCA intervention, MCA patients might also wait a short while for group therapy, not seeing any psychotherapist in that interval. As all participants start group therapy, they will have a designated contact person, but only one preparatory individual therapy session before entering the specific treatment group.

### Intervention fidelity

The intervention will be carried out by the first author, a resident in psychiatry. He has received training and supervision on the assessment battery from national experts in the field. The intervention will be supervised by the third author, who is trained in therapeutic assessment. Audio or video recordings of MCA consultations will be used for supervision purposes and to secure intervention fidelity.

#### Outcomes’ objective 1: Feasibility

- Acceptability: The feasibility criterion for acceptability in patients is supported if 25% of patients who are found eligible for inclusion and who have received formal information (information meeting with the first author) about the trial agree to participate in the trial.
- Satisfaction: The feasibility criterion for satisfaction in patients is supported if the mean CSQ-8 score is ≥ 3 (see below for description of instrument).
- Adherence: The feasibility criteria for adherence in patients is supported if 75% complete the MCA intervention (attend all MCA sessions including feedback-session).
- Time spent on the intervention: We evaluate the resources used to complete the study by recording the time spent in direct contact with the patient used to complete the MCA intervention [48].
- Evaluation of the Intervention Questionnaire (EQ)
- Adverse events

#### Outcomes’ objective 2: Trial outcomes

See Table 1 for a table of measurements.

**Table 1:** Table of measurements CO-ASSM-RCT

	**Baseline (T0)**	**End of intervention (T1)**	**After 4 sessions of group psychotherapy (T2)**
URICA	x	x	
LSAS	x	x	x
RSES	x	x	x
GSES	x	x	x
CSQ-8 ^a^		x	x
PROM	x	x	x
EHR ^a^			x
EQ ^a^		x	
RPI	x	x	

^a^Feasibility outcomes

### Primary outcome

#### University of Rhode Island Change Assessment Scale (URICA)

URICA is a 32-item self-report measure including 4 subscales, designed to quantify the patient’s motivation for change: The four subscales are Pre-contemplation, Contemplation, Action, and Maintenance [60]. We will utilize the Contemplation score as our primary outcome.

### Secondary outcomes

#### The Liebowitz Social Anxiety Scale-Self-Report (LSAS)

The self-administered 24-item LSAS-SR [60], which is highly correlated with the clinician-administered version [61], includes questions pertaining to social interaction and performance situations. The LSAS-SR has shown to have good convergent, discriminant validity, and reliability [62].

### Rosenberg Self-Esteem Scale (RSES)

The RSES is a 10-item measure of self-esteem that includes five positive items and five negative items which are reverse-scored [63]. In general, the RSES has demonstrated good convergent validity and good test–retest reliability, and in similar populations of adults with social phobia, the RSES has demonstrated high internal consistency [64].

### General Self-Efficacy Scale (GSES)

The GSES is a 10-item psychometric scale that is designed to assess optimistic self-beliefs to cope with a variety of difficult demands in life. In contrast to other scales that were designed to assess optimism, this one explicitly refers to personal agency, i.e., the belief that one’s actions are responsible for successful outcomes [65, 66].

### Exploratory outcomes

#### Readiness for Psychotherapy Index

The RPI is a 42-item self-report measure that uses a 5-point Likert scale to assess 7 dimensions of readiness for psychotherapy: level of distress, desire for change, willingness to work in therapy, recognition of problems as psychological, willingness to discuss personal matters, willingness to endure discomfort in therapy, and responsibility for change [67]. The questionnaire will be translated and validated for use in a Danish mental health service population as part of the present study.

### National Patient-Reported Outcome Measures (PROM)-Psychiatry

The Danish National PROM is a 19-item self-report measure covering patients’ own views on their mental and physical health and level of general well-being [68]. It includes the WHO Well-Being Index (WHO-5), the Work and Social Adjustment Scale (WSAS) [69], and general items from the SF36.

### Data from Electronic Health Records (EHR)

We will monitor the number of no-shows and the number of diagnostic re-classifications by accessing the included patients’ EHR.

### User evaluations

#### Client Satisfaction Questionnaire (CSQ-8)

The CSQ-8 is a self-report questionnaire, constructed to measure satisfaction with services received by individuals and families [70].

### Evaluation of the Intervention Questionnaire (EQ)

This questionnaire focusing on the patient’s and therapist’s evaluation of the intervention, which is purpose-made for the current study, will be distributed at the end of the intervention. Items will be constructed as Likert Scale feedback forms consisting of a list of statements about different aspects of the course of the intervention. Response possibilities are five categories ranging from “very much in agreement” to “not at all.”

### Adverse effects

We expect no serious risks or adverse effects to the included patients. We monitor for adverse events, in particular suicidal behavior/ideation, and will check for this at every visit to the clinic. If a patient is admitted during participation in the study, a senior consultant (RB) will decide whether the patient can continue to participate.

### Patient and public involvement

We will establish a user panel who will help interpret the findings of the study, and we will include the feedback in our reporting of the findings of the present study, and in the preparations for a possible full-scale phase-3 study of the intervention. The user panel will include five patients recruited from the clinics and they will meet two times throughout the study. They will be users of the Danish MHS but not participants in this trial.

#### Statistical considerations

As the primary objective of the present trial is to test feasibility, a formal power calculation is not required. However, the present trial also wishes to examine MCA versus AAU, and we present the following power calculation for the primary outcome: readiness for change. Sample size calculation is based on figures in Dozois et al. [71] of patients with panic/anxiety, where the primary outcome “Contemplation stage score” in readiness for change was mean 37.3 (SD 1.9) for CBT responders and mean 34.4 (std 3.4) for non-responders, i.e., with significance level 5% and power 90%. This yields a total sample of 36 with 18 patients in each arm. We strive for 42 patients to account for attrition around 15%. We find it feasible to include the 42 patients since 64 patients are offered treatment packages annually (40 SP and 24 AvPD).

A detailed statistical analysis plan will be published on ClinicalTrials.gov prior to data processing initiation. Briefly, we expect to report descriptive data such as data for the feasibility criterion as percentages, means, and variation as standard deviation. Outcomes will be analyzed as continuous and categorical measures (i.e., responder status and new diagnosis, see below). All statistical analyses will be performed as intention-to-treat analyses (ITT); however, main outcomes will also be analyzed for completers only for sensitivity reasons. Missing data will be handled by the use of multiple imputations. The pre- to post-treatment effects of the MCU or AAU will be determined utilizing a series of repeated measures analyses of variance (rmANOVAs). Analysis will be based on masked data (recoded data for concealment of allocation). Conclusions of the main outcomes will be formulated prior to unmasking the data. Statistical calculations will be performed prior to unblinding. All statistical calculations will be performed in R and RStudio [72].

### Dissemination policy

The results of the present study will be disseminated by the Psychiatric Research Unit social media account and website. We will also seek to publish it through high-impact international peer-reviewed journals and present it at conferences for clinicians, commissioners, and researchers working in the mental health field. Both negative and positive findings will be published. The trial is registered at ClinicalTrials.gov (Nr 2,021,001). Although steps will be taken to avoid it, protocol deviations may happen. Protocol deviations that occur after the start of trial recruitment will be communicated at https://clinicaltrials.gov and detailed in publications.

### Trial status

The trial began recruitment in October 2021, and the last participant is expected to be included in January 2023. The experimental intervention ran from November 2021 to April 2023.

## Discussion

The current study will be the first RCT investigating MCA in a mental health service setting. It will be a feasibility study and will test the study hypothesis in a small clinical sample. If the present study is successful, it may be followed up by other, larger clinical studies on MCA. The study will contribute to sparse existing research concerning the impact of clinical assessment; it will provide important new knowledge about the effects of routine and systematic patient-centered clinical assessment and generate effect size measures for future power calculations. It will also generate data regarding patients’ readiness for psychotherapy, and the percentage of patients who are wrongly diagnosed in a prototypical Danish public psychotherapeutic healthcare clinic.

We believe that the intervention will have a positive effect on the included patients. However, there is a risk that the patients receiving MCA may not benefit from the excess assessment but that the treatment will instead increase dropout due to the patient becoming overwhelmed. There is also a possibility of the patients becoming upset or disappointed due to the new knowledge they receive about themselves. Ultimately, the MCA may yield an unexpected diagnosis which could severely change the way the patient sees herself and the way society in general sees the patient. Many of these problems may, however, also occur in AAU. We will evaluate this by comparison of dropout from MCA and AAU.

If the current project documents the feasibility of the approach, further studies should examine the incremental value of MCA on patient outcome in terms of treatment completion or number of treatment sessions attended, and cost-effectiveness.

By the end of the present project, we will be able to decide whether the results are sufficiently promising to pursue a full trial (phase III) [73]. For that purpose, the study output also encompasses the development of a MCA protocol for clinicians and an adjoining fidelity instrument.

## Data Availability

The datasets generated by the planned study will not be publicly available due to the rules of the Danish Data Protection Agency but will be available from the corresponding author, after publication, on reasonable request and following a signed confidentiality agreement with PI and the Danish Data Protection Agency Region Zealand.
